# Carbonation of Ammonium Diuranate Filtrate to Enhance Uranium Rejection by Nanofiltration

**DOI:** 10.3390/membranes15050133

**Published:** 2025-05-01

**Authors:** Runci Wang, Zhongwei Yuan, Xiang Meng, Taihong Yan, Weifang Zheng

**Affiliations:** Department of Radiochemistry, China Institute of Atomic Energy, Beijing 102413, China

**Keywords:** uranium, nanofiltration, ammonium diuranate filtrate, concentration polarization, membrane separation

## Abstract

A commercial polymeric nanofiltration membrane (NF270, DuPont) was employed for uranium removal from ammonium diuranate filtrate (ADUF). Carbonate supplementation through ammonium carbonate addition enhanced uranium rejection via formation of uranyl–carbonate coordination complexes. Systematic speciation analysis of uranium species in ADUF was conducted, coupled with calculation of the concentration polarization modulus to optimize ammonium carbonate dosage. The experimental results demonstrated that with 680 mg/L ammonium carbonate addition, the permeate uranium concentration decreased from 1.2 mg/L to 0.64 mg/L. This study confirms the technical feasibility of ADUF carbonation pretreatment for improving uranium retention efficiency in nanofiltration processes, achieving 46.7% reduction in uranium permeation flux.

## 1. Introduction

The ammonium diuranate (ADU) precipitation process plays a critical role in uranium purification and conversion. In this process, ammonia is introduced into purified uranyl nitrate solutions to precipitate ADU [1]. Subsequent filtration generates ammonium diuranate filtrate (ADUF), a low-level radioactive waste containing substantial ammonium nitrate (30–40 g/L) and trace uranium (mg/L levels). Traditional uranium recovery from ADUF using silica gel in China produces significant secondary waste, necessitating alternative approaches.

Membrane separation technologies have emerged as promising solutions for uranium removal in nuclear applications [2]. While reverse osmosis (RO) has been attempted for ADUF treatment [3], its operational limitations include high hydraulic pressures and severe flux decline due to elevated osmotic pressures [4]. Forward osmosis (FO), though operable at lower pressures [5], exhibits substantially reduced flux compared to RO [6], limiting its utility to small-scale operations. Nanofiltration (NF) presents distinct advantages for selective separation of divalent ions and larger molecules from monovalent species [7], with demonstrated ammonium nitrate rejection rates of 16–19% compared to RO’s 34–85% [8]. Our previous studies revealed that NF270 membranes (DuPont-FilmTec) achieve 97% uranium rejection while maintaining < 10% ammonium nitrate retention in ADUF [9,10]. However, similar to uranium rejection from seawater, high-salinity environments can compromise uranium rejection through electrical shielding effects [9].

Complexation strategies using additives like EDTA and sodium sulfate have shown potential for improving uranium rejection [3], but introduce impurities, complicating downstream uranium reuse. Carbonate ions offer a non-contaminating alternative, as evidenced by Elizabeth et al. [10], who achieved 94% uranium rejection using SW30 NF membranes (Dow/Brazil) for ammonium uranyl carbonate filtrate (AUCF) treatment containing 1.6 g/L carbonate and 300 g/L ammonium nitrate. The superior performance stems from formation of the highly charged UO_2_(CO_3_)_3_^4−^ complex, which combines enhanced charge repulsion and size exclusion mechanisms; this phenomenon is also observed in natural water uranium removal via NF [11,12,13].

This study proposes carbonate modification of ADUF to promote uranyl carbonate complexation, thereby improving NF performance. Considering the critical C/U molar ratio (CO_3_^2−^:UO_2_^2+^) identified in AUCF uranium recovery studies [14,15], we systematically investigated uranium speciation under varying carbonate concentrations. Furthermore, we accounted for concentration polarization (CP) effects at the membrane surface [16], which can exacerbate fouling [17] and necessitate correction of carbonate dosage via concentration polarization modulus calculations [18].

The objectives of this work were threefold: (1) to characterize uranium speciation in carbonate-modified ADUF, (2) to quantify CP effects using the velocity variation method [19], and (3) to evaluate uranium rejection enhancement through controlled carbonate addition. Simulated ADUF solutions (uranyl nitrate/ammonium nitrate mixtures) were employed throughout this investigation.

## 2. Experimental Section

### 2.1. Materials

The NF270 polymeric nanofiltration membrane (DuPont) was employed based on its previously demonstrated suitability for ADUF treatment [20,21].

Simulated ADUF solutions were prepared by dissolving ammonium nitrate (NH_4_NO_3_, AR, Beijing Chemical Plant, Beijing, China) and uranyl nitrate hexahydrate (UO_2_(NO_3_)_2_·6H_2_O) in demineralized water (conductivity = 0.7–0.8 μS cm^−1^), achieving final concentrations of 35 g L^−1^ NH_4_NO_3_ and 40 mg L^−1^ uranium.

### 2.2. Experimental Setup and Methods

**Figure 1:**
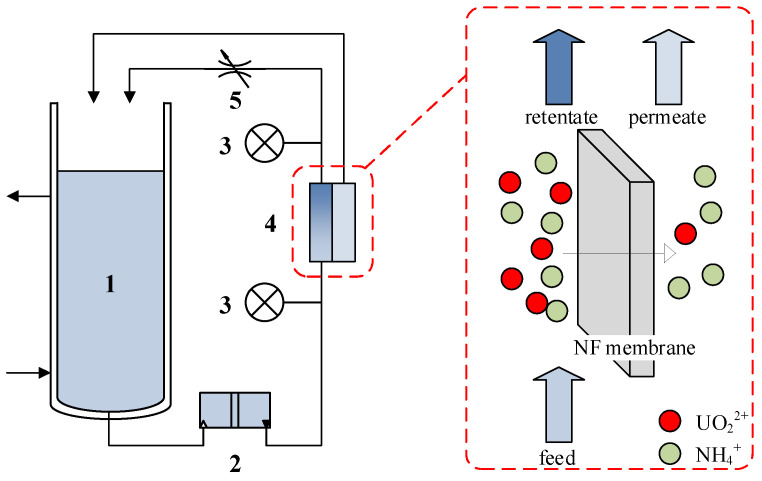
Scheme of the NF experimental setup. 1. Feed tank. 2. Diaphragm pump. 3. Pressure gauge. 4. Flat-sheet membrane cell. 5. Pressure control valve.

The cross-flow nanofiltration system (Figure 1) comprised a 2 L feed tank equipped with temperature-controlled recirculation (25 ± 1 °C), a diaphragm pump, a digital pressure transducer (0–3 MPa range), a flat-sheet membrane test cell (70 cm^2^ effective filtration area), and a precision pressure regulation valve. Both permeate and retentate streams were continuously recirculated to maintain stable feed composition. System operation maintained transmembrane pressures of 1.0–2.0 MPa with tangential flow velocities ranging from 10 to 50 cm·s^−1^.

Prior to experimental trials, the NF270 membrane was subjected to compaction under controlled transmembrane pressure conditions by demineralized water. A hydraulic pressure of 3.0 MPa was applied for a minimum duration of 60 min, until the permeate flux variation coefficient reached ≤1% over 15 min.

Uranium concentration analysis was performed using inductively coupled plasma mass spectrometry (ICP-MS; ELAN DRC-e, PerkinElmer, Waltham, MA, USA). Ammonium nitrate concentrations were determined through conductivity measurements (DDSJ-308A conductivity meter, Shanghai Leici Instrument Co., Shanghai, China) based on their predominant ionic conductivity contribution.

The rejection coefficient (R) was calculated according to the standard membrane performance equation:

(1) $$R=(1-c_{p}/c_{b})$$

where $c_{p}$ is the concentration of permeate and $c_{b}$ the concentration of bulk feed.

## 3. Results and Discussion

### 3.1. Uranium Speciation Analysis

**Figure 2:**
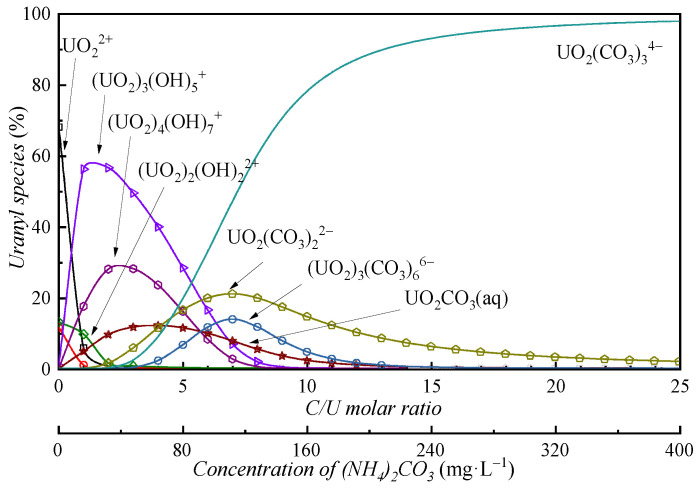
Uranyl species in simulated ADUF as a function of the number of carbonate ions evaluated with Visual MINTEQ.

Previous studies established uranium speciation in carbonate-free ADUF [21]. In this work, we systematically investigated uranium speciation variations with carbonate addition using Visual MINTEQ (version 3.1), an equilibrium speciation model for aqueous systems. Figure 2 illustrates the calculated uranium species distribution in simulated ADUF.

In the absence of carbonate ions, UO_2_^2+^ constituted the dominant species (>85%). Gradual carbonate addition induced sequential speciation shifts: the UO_2_^2+^ fraction decreased precipitously as hydrolyzed species and carbonate complexes emerged. Notably, substantial uranyl hydrolysis occurred at C/U = 0–5, mirroring ADU precipitation dynamics. Uranyl carbonate complexes became predominant only when carbonate concentrations significantly exceeded uranium levels, with UO_2_(CO_3_)_3_^4−^ accounting for >90% of species at C/U ≥ 13. This behavior aligns with the carbonate–hydroxide competition mechanism governing uranyl complexation [14].

**Figure 3:**
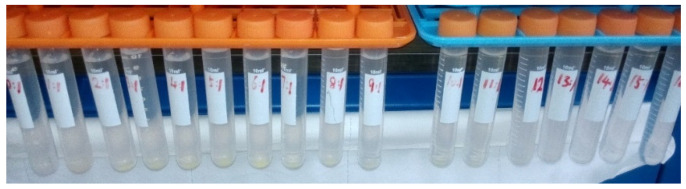
The simulated ADUF with C/U molar ratio = 1~16 from left to right.

The conclusions above were supported by experiments (see Figure 3). Different amounts of ammonium carbonate were added into simulated ADUF and allowed to stand for over 1 h. Yellow precipitates formed at the bottom of the tubes with C/U = 1~12. No precipitates appeared when the ratio was 0 or ≥13.

### 3.2. Concentration Polarization

**Figure 4:**
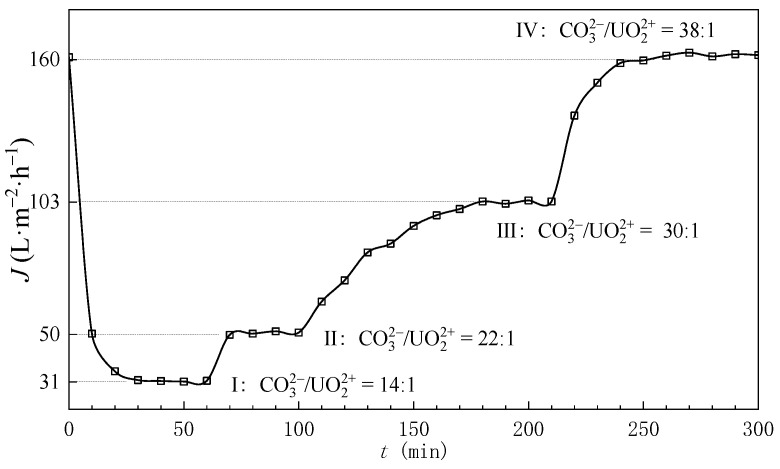
The flux of permeate with different mole ratios of CO_3_^2−^ and UO_2_^2+^; the transmembrane pressure is 2.0 MPa and tangential velocity is 40 cm/cm.

The theoretical calculations in Section 3.1 suggested a required bulk C/U molar ratio of 13 for effective uranyl carbonate complexation (>90%). However, experimental observations revealed a critical deviation. During NF experiments, ammonium carbonate solutions were incrementally introduced into the ADUF feed solution to elevate bulk C/U ratios, with permeate flux being periodically monitored. The results demonstrated that permeate flux exhibited a persistent decline upon the initial introduction of carbonate species. Subsequent continuation of carbonate dosing induced gradual flux recovery, with full flux recovery being achieved at C/U = 38 (Figure 4). This operational threshold implied membrane fouling through uranyl hydrolysis when C/U < 38, necessitating reevaluation of the theoretical framework.

The discrepancy originates from concentration polarization (CP) effects. The CP modulus $c_{m}/c_{b}$, defining the membrane surface-to-bulk concentration ratio, is governed by [18]:

(2) $$\frac{c_{m}}{c_{b}}=\frac{\exp(J/k)}{R_{int}+(1-R_{int})\exp(J/k)}$$

where $c_{m}$ is the concentration at the membrane surface, $c_{b}$ is the concentration in the bulk feed, J is the flux, $R_{int}=(1-c_{p}/c_{m})$ is the intrinsic retention, and k is the mass transfer coefficient, i.e., as seen in [21]:

(3) $$k=D/\delta =a{v_{t}}^{1/3}$$

where D is the diffusion coefficient, δ is the thickness of the concentration polarization layer, $a=1.85{d_{h}}^{-1/3}L^{-1/3}D^{2/3}$, $d_{h}$ and L are hydraulic diameter and length of the fluid channel of the experimental setup, and $v_{t}$ is the tangential velocity.

**Figure 5:**
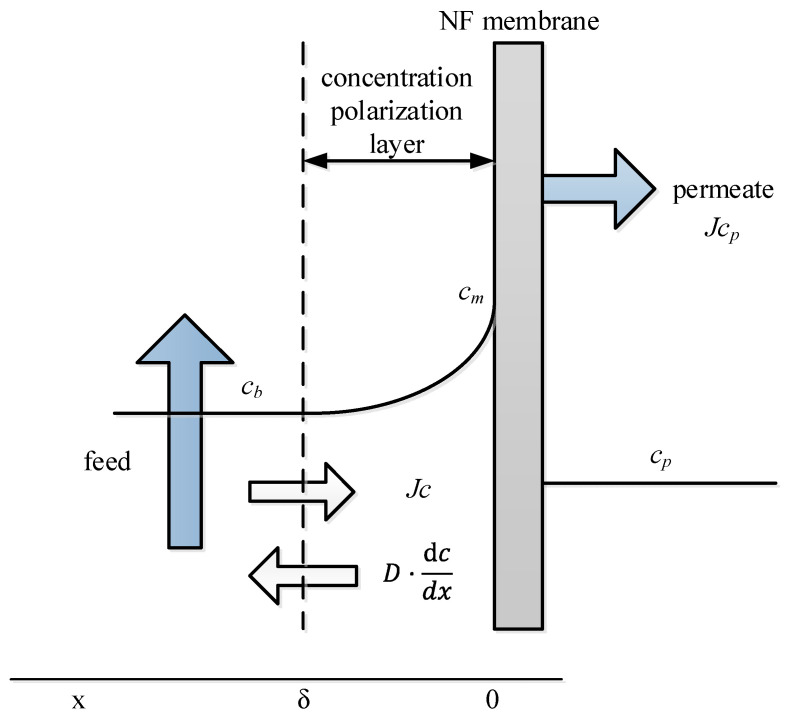
Scheme of concentration polarization [18].

The concentration polarization modulus $c_{m}/c_{b}$ can be determined by NF experiments. As shown in Figure 5 in steady-state conditions,

(4) $$Jc+D\cdot \frac{dc}{dx}=Jc_{p}$$

and ${\left.c\right|}_{x=0}=c_{m}$, ${\left.c\right|}_{x=\delta }=c_{b}$. Integration of Equation (4) results in

(5) $$\frac{c_{m}-c_{p}}{c_{b}-c_{p}}=\exp(\frac{J}{k})$$

By subscribing $R=(1-c_{p}/c_{b})$ and $R_{int}=(1-c_{p}/c_{m})$ into Equation (5), we obtain

(6) $$\frac{R_{int}/(1-R_{int})}{R/(1-R)}=\exp(\frac{J}{k})$$

And by subscribing Equation (3) into Equation (6), we obtain

(7) $$ln\left(\frac{R}{1-R}\right)=ln\left(\frac{R_{int}}{1-R_{int}}\right)-\frac{1}{a}\cdot J\cdot {v_{t}}^{-1/3}$$

**Figure 6:**
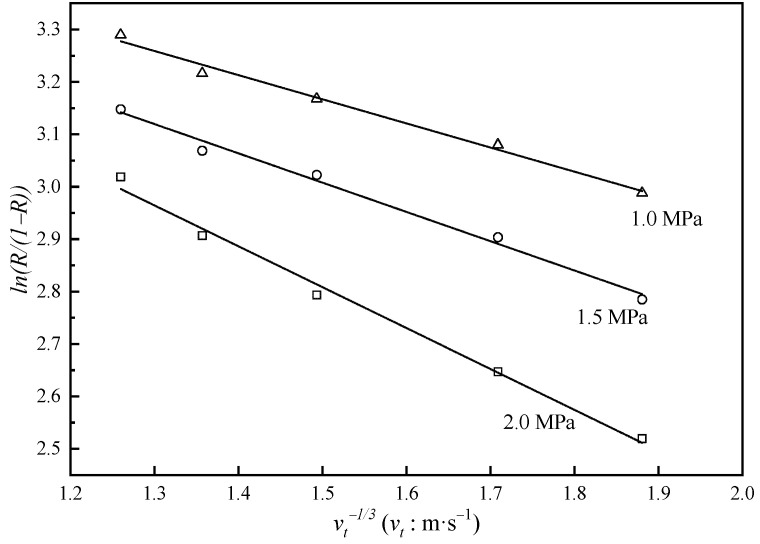
Relationship between $ln\left(\frac{R}{1-R}\right)$ and ${v_{t}}^{-1/3}$. R is the rejection of uranium. Markers: experimental data, solid lines: data calculated with Equation (7).

**Figure 7:**
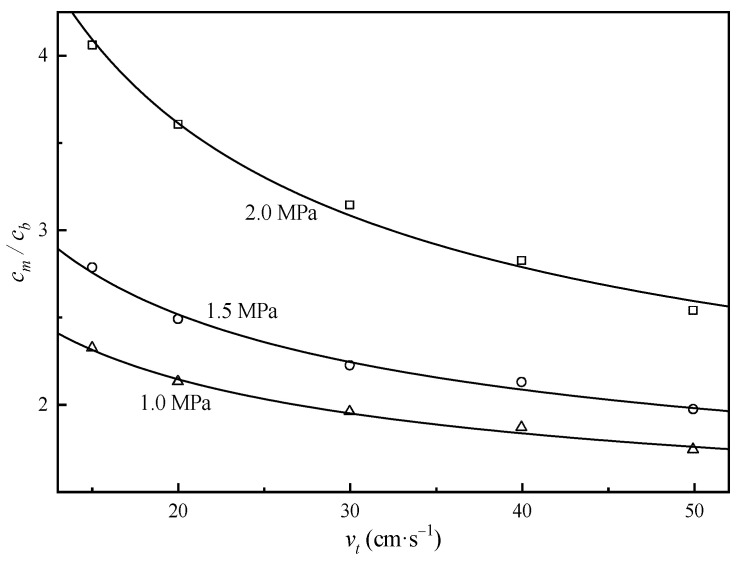
Concentration polarization modulus of uranium with different tangential velocities and transmembrane pressures. Markers: experimental data, solid lines: data calculated with Equation (2).

Based on the data obtained through NF experiments, we could obtain both $R_{int}$ and a by linearly fitting $ln\left(\frac{R}{1-R}\right)$ and ${v_{t}}^{-1/3}$. The result is shown in Figure 6, and $R_{int}=97.9\sim 98.2\%$, $a=3.45\sim 4.32\times 10^{-5}m^{2/3}\cdot s^{-2/3}$. Then, we entered a into Equation (3) to calculate the mass transfer coefficient k and finally entered $R_{int}$ and k into Equation (2) to obtain the concentration polarization modulus $c_{m}/c_{b}$ (see Figure 7).

As demonstrated in Figure 7 and Equation (2), the concentration polarization modulus of uranium exhibited an inverse correlation with tangential flow velocity ($v_{t}$) and a positive dependence on transmembrane pressure. Under the experimental conditions specified in Figure 4, the calculated uranium concentration polarization modulus reached 2.8 (see Figure 6). This calculation indicates that the required CO_3_^2−^/UO_2_^2+^ molar ratio (C/U) in bulk solution must satisfy 13×2.8=36.4 to ensure the effective C/U ratio at the membrane surface attains the threshold value of 13. Note that the concentration polarization modulus for carbonate ions was assumed as 1 based on its lower retention coefficient.

While this estimation approach contains inherent simplifications, the derived values demonstrated strong consistency with experimental NF performance data. Crucially, these results quantitatively establish that the bulk-phase C/U ratio must exceed the membrane surface requirement by a factor equivalent to the uranium concentration polarization modulus. To account for potential flux variations and prevent membrane fouling, we implemented a safety margin (42) in subsequent experiments, corresponding to ammonium carbonate addition of 680 mg L^−1^.

### 3.3. Rejection of Uranium in ADUF

**Figure 8:**
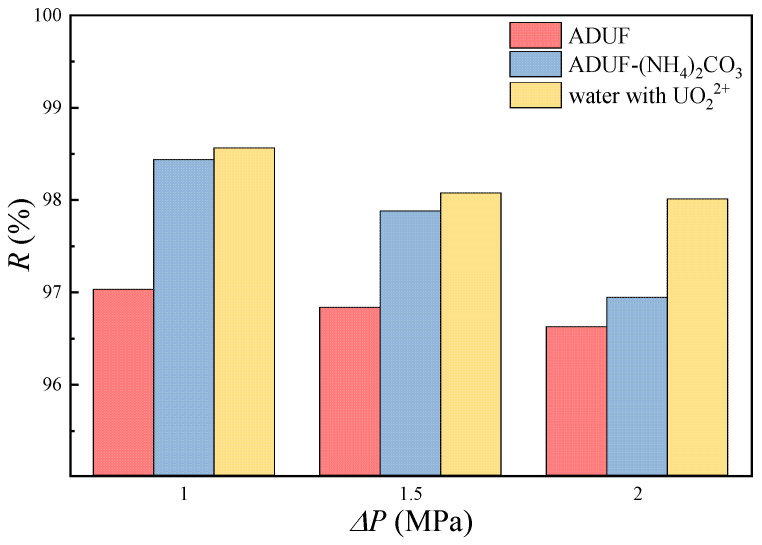
The rejection of uranium in ADUF, ADUF dissolving CO_3_^2−^ and CO_3_^2−^/UO_2_^2+^ = 42, and demineralized water dissolving 40 mg/L uranium. The tangential velocity was 40 cm/s.

The rejection of uranium by NF is shown in Figure 8. The data demonstrate that adding ammonium carbonate to ADUF significantly increased the rejection of uranium. The rejection of uranium increased from 97.0% to 98.4% at 1.0 MPa. The absolute increase in rejection may appear limited, but the permeate concentration of uranium was decreased by 46.7% (from 1.2 mg/L to 0.64 mg/L), effectively halving uranium leakage.

We can also find some more conclusions. In deionized water containing UO_2_^2+^, the rejection reaches 98.5% at 1 MPa. According to Marchenko’s report [22], the uranium–oxygen distances in hydration shells are 2.42 Å for UO_2_^2+^ and 4.19 Å for UO_2_(CO_3_)_3_^4−^. Given the NF270 membrane’s pore radius range of 4.2~4.4 Å [23,24], steric exclusion is likely the dominant retention mechanism for uranium [13]. But it must be noted that the rejections in ADUF were significantly lower than those in water, and a similar phenomenon is also reported in other papers [25,26]. This suggests that 35 g/L ammonium nitrate in ADUF induces charge shielding on NF membrane surfaces, thereby reducing electrostatic repulsion. Due to the fact that the hydration radii of UO_2_^2+^ and UO_2_(CO_3_)_3_^4−^ are not significantly larger than the membrane pore radius, and the experimental results show a strong charge shielding phenomenon, we propose that charge interaction dominates over steric effects in governing uranium retention.

We can also determine from Figure 8 that the rejection is higher at a lower transmembrane pressure, and this phenomenon can be explained by the concentration polarization phenomenon. Lower transmembrane pressure resulted in reduced permeate flux, ultimately leading to a decreased concentration polarization modulus, as described in Equation (2). This means that the membrane surface concentration $c_{m}$ was reduced at a lower transmembrane pressure. Since the intrinsic retention $R_{int}=(1-c_{p}/c_{m})$ remained essentially constant during NF experiments, lower $c_{m}$ values produced proportionally lower $c_{m}$ values, thereby increasing the observed rejection $R=(1-c_{p}/c_{b})$. The functional relationship between observed rejection and transmembrane pressure can be mathematically expressed as

(8) $$R={\left[1+\left({R_{int}}^{-1}-1\right)\exp\left(\frac{L\Delta P}{k}\right)\right]}^{-1}$$

where L is the membrane permeability, ΔP is the transmembrane pressure, and J=LΔP. Equation (8) can be derived from Equation (6).

## 4. Conclusions

The uranium rejection efficiency of nanofiltration (NF) was significantly enhanced following the introduction of carbonate ions, suggesting that carbonation of ADUF could represent a viable strategy for improving uranium retention performance. The optimal ammonium carbonate dosage demonstrated dependence on the concentration polarization characteristics of the NF system configuration. Under experimental conditions employing 680 mg/L ammonium carbonate, the permeate uranium concentration exhibited a notable reduction from 1.2 mg/L to 0.64 mg/L.

However, the elevated ammonium nitrate concentration in ADUF was found to neutralize membrane surface charges, consequently diminishing rejection efficiency despite carbonate supplementation. Current technical constraints prevent the implementation of tighter separation membranes like reverse osmosis (RO) membranes due to prohibitive osmotic pressure limitations. Therefore, a hybrid NF-RO membrane configuration shows promise for enhanced ADUF treatment efficacy, with systematic investigation of this approach presently being conducted.

## Data Availability

The original contributions presented in this study are included in the article. Further inquiries can be directed to the corresponding author.
